# Odor coding in the mammalian olfactory epithelium

**DOI:** 10.1007/s00441-020-03327-1

**Published:** 2021-01-06

**Authors:** Smija M. Kurian, Rafaella G. Naressi, Diogo Manoel, Ann-Sophie Barwich, Bettina Malnic, Luis R. Saraiva

**Affiliations:** 1 Sidra Medicine, Doha, Qatar; 2 Department of Biochemistry, University of São Paulo, São Paulo, Brazil; 3 Indiana University, Bloomington, IN USA; 4 Monell Chemical Senses Center, Philadelphia, USA; 5 College of Health and Life Sciences, Hamad Bin Khalifa University, Doha, Qatar

**Keywords:** Olfaction, Odor coding, Combinatorial code, Odorant, Receptor, Antagonist, Smell

## Abstract

Noses are extremely sophisticated chemical detectors allowing animals to use scents to interpret and navigate their environments. Odor detection starts with the activation of odorant receptors (ORs), expressed in mature olfactory sensory neurons (OSNs) populating the olfactory mucosa. Different odorants, or different concentrations of the same odorant, activate unique ensembles of ORs. This mechanism of combinatorial receptor coding provided a possible explanation as to why different odorants are perceived as having distinct odors. Aided by new technologies, several recent studies have found that antagonist interactions also play an important role in the formation of the combinatorial receptor code. These findings mark the start of a new era in the study of odorant-receptor interactions and add a new level of complexity to odor coding in mammals.

## Introduction

*“I should think we might fairly gauge the future of biological science, centuries ahead, by estimating the time it will take to reach a complete, comprehensive understanding of odor. It may not seem a profound enough problem to dominate all the life sciences, but it contains, piece by piece, all the mysteries.” — Lewis Thomas.*

Smelling starts with a sniff. The process of breathing in air into the nose floods the nasal cavity with myriad odorous molecules, or simply put, odorants. These molecules may smell pleasant, repulsive, or act as carriers of critical biological or ecological messages.

Odorants communicating vital biological information typically elicit behavioral and physiological changes in animals, thus playing a pivotal role in the survival and the propagation of the species (Li and Liberles, 2015). In some cases, the same odorant delivers different biological messages to animals of different species. In others, the identity of these ecologically-relevant odorants may vary greatly among different species, ultimately driving evolutionary adaptations to distinct ecological niches (Bear, et al., 2016; Li, et al., 2013; Manoel, et al., 2019).

A major challenge in studying smell and odor-guided behaviors has been the understanding of the biological mechanisms that enable the discrimination of a large number of odor cues, which are typically presented to the animal’s nose in virtually infinite combinations of mixtures and concentrations.

This review presents a brief historical description of the key findings and early challenges surrounding odor coding in the mammalian nose. It discusses how recent advances in olfactory neurobiology fundamentally inform our understanding of the interactions between odorants and their receptors in the nose, and how this knowledge impacts theories of odor perception.

## Organization of the mammalian olfactory system

The peripheral olfactory system of most mammalian species involves two major olfactory organs: the olfactory mucosa (OM) located at the top of the nasal cavity and the vomeronasal organ (VNO) sitting at its base (Buck, 2012). The anatomical structure of the olfactory system can vary significantly between species, with some mammalian lineages (e.g., catarrhine monkeys, apes, and humans) lacking a VNO (Keverne, 1999), and other species (e.g., rodents) displaying additional olfactory organs, such as the septal organ of Masera and the Grueneberg ganglion (Barrios, et al., 2014; Ma, 2010).

**Fig. 1:**
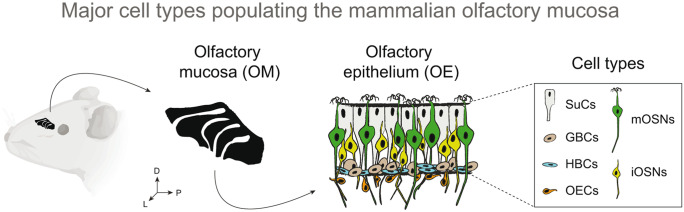
The major cell types of the mammalian olfactory mucosa (OM). In mammals, the OM is composed by the olfactory epithelium (OE) and a submucosa. The OE is a pseudostratified epithelium composed mainly by sustentacular cells (SUCs), globose basal cells (GBCs), horizontal globose cells (HBCs), immature olfactory sensory neurons (iOSNs) and mature olfactory sensory neurons (mOSNs). The olfactory ensheathing cells (OECs) are an important cell type populating the submucosa

Our focus is on the OM of the nose, which is composed of the olfactory epithelium (OE) and a submucosa. The OE is mainly populated by sustentacular cells, horizontal and globose basal cells, immature and mature olfactory sensory neurons (Fig. 1). The submucosa sitting below contains olfactory ensheathing cells, glandular and cavernous tissues, blood, and lymph vessels (Cuschieri and Bannister, 1975; Huard, et al., 1998; Morrison and Costanzo, 1992; Sharma, et al., 2019). Odorant reception occurs primarily in the OE via the mature olfactory sensory neurons (hereafter referred to as OSNs). This cell type, with its molecular and physiological architectures, thus is at the center of this review.

## Early challenges in understanding odor coding and the discovery of the odorant receptors

*“the olfactory imprint is collected in the mucosa by the peripheral expansion of the bipolar cells and is then transferred to the glomeruli where […] cells from the molecular layer collect said imprint to raise it to the brain.”*

*- Santiago Ramón y Cajal*

The two pioneers of neuroscience (and eternal rivals), Camillo Golgi and Santiago Ramón y Cajal, had described the basic neuroanatomical structure of the olfactory system in the late nineteenth century (Golgi, 1875, Ramón y Cajal, 1892). However, the concept of odorant receptors was considered only mid-twentieth century (Jones and Jones, 1953; Ottoson, 1954; Pauling, 1946; Skouby and Zilstorff-Pedersen, 1954; Sviridenko, 1951), while the genes encoding odorant receptors remained incognito for almost the entire twentieth century.

The foundation for discovering the receptors genes was laid in the 1970s and ‘80 s, with an increase in molecular studies that suggested a second messenger mechanism in olfaction. First, high adenylate cyclase activity was found in olfactory ciliary preparations of dissociated frog OSNs (Kurihara and Koyama, 1972; Pace, et al., 1985), a biochemical finding later confirmed physiologically (Firestein, et al., 1991). This data was followed by the identification of cyclic AMP (cAMP) as the secondary messenger in olfactory reception (Gesteland, 1976; Minor and Sakina, 1973). Contemporary technological developments, such as electrophysiological recordings, revealed that distinct odorants evoke distinguishable activation patterns in the OE (Kauer and Moulton, 1974; Mackay-Sim, et al., 1982), and even suggested the existence of multiple OSNs subtypes (Gesteland, 1976; Holley and MacLeod, 1977; Lancet, 1986; Sicard, 1985; Sicard and Holley, 1984).

Towards the end of the 1980s, mounting evidence pointed to G-protein coupled receptors (GPCRs) as the strongest candidates for odorant receptors (Lancet, 1986). Especially the identification of an olfactory-specific gene coding for a Gα protein (Gα_olf_) and for a nucleotide-gated channel indicated that odorant activation involved G-protein mediated production of cAMP (Dhallan, et al., 1990; Jones and Reed, 1989). Around the same time, experiments using dissociated newt OSN provided evidence of intracellular calcium signaling during odorant binding. These experiments further implied a link to the mechanism of adenylate cyclase or gating of ion channels (Kurahashi and Shibuya, 1990).

The path looked paved for the discovery of the odorant receptors. Still, their identification as GPCRs would take several more years before transforming the field (Buck and Axel, 1991; Firestein, et al., 2014). Notable about this discovery was Buck's ingenious experimental design, which revealed a crucial feature of the OR family that would expand understanding of GPCRs: the mosaic character of the OR multigene family. ORs are highly conserved throughout evolution while also exhibiting striking structural diversity across their members. Instead of being defined by a specific set of shared amino acid sequences, the OR family relation is cross-cutting, meaning members share different sequences with various other members.

The mosaic character of OR genes had also made their discovery impracticable (Barwich, 2020; Buck, 2004). The standard discovery method of new gene families at the time was PCR. However, the amplification of genetic material with the known GPCR primer pair failed. Buck's use of RNA instead of DNA in combination in tandem with her design of 11 degenerate primers, amplifying related but not identical sequences (based on Buck's interest in genetic diversification), yielded the jackpot. Mammalian odorant receptors turned out to be the largest multigene family in the mammalian genome, containing ~ 400 intact genes in humans, ~ 1100 in mouse and ~ 2000 in elephants (Godfrey, et al., 2004; Malnic, et al., 2004; Niimura, et al., 2014; Zhang and Firestein, 2002).

## Other chemosensory receptors in the OE

Since the discovery of the OR gene family in 1991, other evolutionary conserved families of chemosensory receptor genes were found to be expressed in the mammalian OE, including the trace-amine associated receptors (TAARs), two guanylyl cyclases (GUCY2D and GUCY1B2), and the membrane spanning 4-pass A (MS4A) receptors (Bear, et al., 2016; Fulle, et al., 1995; Greer, et al., 2016; Horowitz, et al., 2014; Leinders-Zufall, et al., 2007; Liberles and Buck, 2006; Omura and Mombaerts, 2015; Saraiva, et al., 2015b, 2019). These ‘atypical’ receptors feature in other reviews in this issue, or were recently covered in other review articles.

## Odorant receptor expression in the OE

**Fig. 2:**
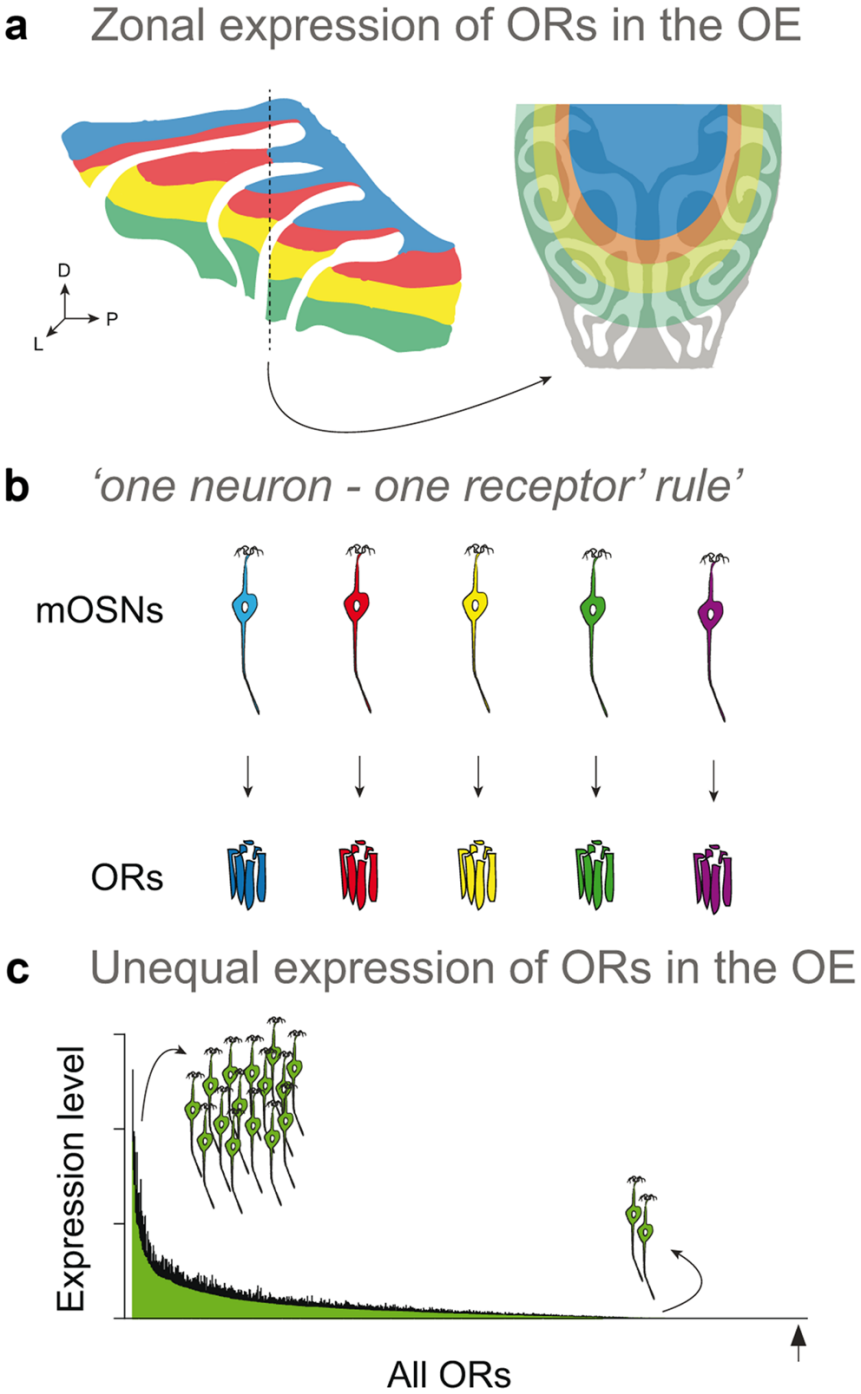
Expression patterns of odorant receptors (ORs) in the olfactory epithelium (OE). (**a**) In the nose, mature olfactory sensory neurons (mOSNs) expressing the same OR gene are stochastically distributed within a spatially restricted area of the OE, also known as a ‘*zone*’. Early studies identified 4 non-overlapping OR expression *zones*, but later studies identified as many as 9–12 partially overlapping *zones*. In the schematic, the 4 non-overlapping OR expression zones (blue, red, yellow, and green colors) are shown: left panel, lateral view of the olfactory mucosa (OM); right panel, a coronal section of the OM (including the respiratory epithelium, in grey). (**b**) In the nose, each mOSN expresses one allele of a single OR gene. This type of expression became known in the field as the ‘*one neuron – one receptor rule*’. (**c**) Recent RNA-seq experiments showed that most intact ORs are expressed in the OE across a large dynamic range, with only a minority being expressed at very high levels. As measured by RNA-seq, the abundance level of a given ORs in the OE correlates perfectly to the number of OSNs expressing it. The arrow depicts the position of the last OR plotted.

Following the discovery of the ORs, Buck and Axel’s laboratories deepened research into OR genetics and wiring. One topic of main interest were the expression patterns exhibited by this remarkable gene family in the OE. By performing RNA *in situ* hybridization experiments, they found that OSNs expressing the same OR gene are randomly distributed within spatially restricted zones in the OE (Fig. 2a) (Ressler, et al., 1993; Vassar, et al., 1993). Other studies confirmed the existence of different spatial patterns of expression, or zones, for mammalian ORs. However, the exact number of zones in the OE and their physiological function remains in debate (Bashkirova, et al., 2020; Coppola, et al., 2019; Horowitz, et al., 2014; Miyamichi, et al., 2005; Tan and Xie, 2018; Zapiec and Mombaerts, 2020). In this context, one possible hypothesis is that the spatial organization of the OE contributes to the maximization of the discriminatory capacity of the peripheral olfactory system (Ressler et al. 1993). Notably, the patterns of odorant sorption in the mouse nose have been found to correlate with the spatial response patterns of OSNs – this association is known as the ‘*sorption hypothesis*’ in olfaction (Scott, et al., 2014), and this idea was even proposed prior to the OR discovery as the ‘*chromatographic hypothesis’* by Maxwell Mozell (Mozell 1966). While this hypothesis has recently been challenged (Coppola, et al., 2019), other studies not only support it (reviewed in (Secundo, et al., 2014)) but also suggest that it could help provide a functional logic underlying the spatial organization of ORs/OSN subtypes in the mouse olfactory epithelium (Ressler, et al., 1993). Future large-scale experiments focused on connecting the zonal expression patterns for all mouse ORs to the physicochemical descriptors of their respective agonists will be critical to stress test this hypothesis.

Another significant breakthrough in understanding how OR genetics determine the wiring of the olfactory pathway arrived with two additional studies. These studies revealed that each OSN expresses only one allele of a single OR gene (Chess, et al., 1994; Malnic, et al., 1999). These findings led to the ‘*one neuron – one receptor rule*’ (Fig. 2b), and several subsequent studies provided additional support to the monogenic and monoallelic expression of OR genes in the OE (Li, et al., 2004; Serizawa, et al., 2003; Shykind, et al., 2004; Tian and Ma, 2008; Tietjen, et al., 2005, 2003). More recently, studies using single-cell RNA-sequencing (RNA-seq) have shown that while immature OSNs express low levels of multiple OR genes, the vast majority of mature OSNs express a single OR gene at high levels (Hanchate, et al., 2015; Saraiva, et al., 2015b; Tan, et al., 2015). These results indicate that singular OR gene expression is achieved during the differentiation of the OSN, and further contributes to understanding the mechanisms of OR gene choice (Nagai, et al., 2016).

Another notable feature of ORs, is that their expression level in the OE can differ dramatically, with some receptors being up to 300-fold more abundant than others (Bressel, et al., 2016; Khan, et al., 2011; Rodriguez-Gil, et al., 2015; Young, et al., 2003; Zhang, et al., 2004). In line with these results, recent RNA-seq studies performed in the OE of several mammalian species (mouse, rat, dog, marmoset, macaque, and human) have shown that the vast majority of ORs (up to 98.9% in mouse) are expressed across a large dynamic range of abundance in the OE, and that OR gene expression levels correlates with the number of OSNs expressing the same OR (Fig. 2c) (Ibarra-Soria, et al., 2014, 2017; Saraiva, et al., 2015a, 2015b, 2019). These studies also indicated that RNA-seq is not only a highly sensitive technique to detect mRNA from ORs, but it also serves as an accurate high-throughput tool to catalog OSN subtype diversity.

## A solution to a number’s problem – the ‘*combinatorial receptor code*’

The total number of stimuli recognized and discriminated by the olfactory system remains to be determined. A recent study estimated that humans can discriminate *at least *1.72 trillion odors (Bushdid, et al., 2014). This number was contested and estimated to constitute the upper bound instead (Gerkin and Castro, 2015; Meister, 2015). In either scenario, the numbers of odorants detected and discriminated by the nose vastly exceed the number of intact ORs present in any given species (Niimura, et al., 2014). So, how does the olfactory system solve this multifactorial problem?

**Fig. 3:**
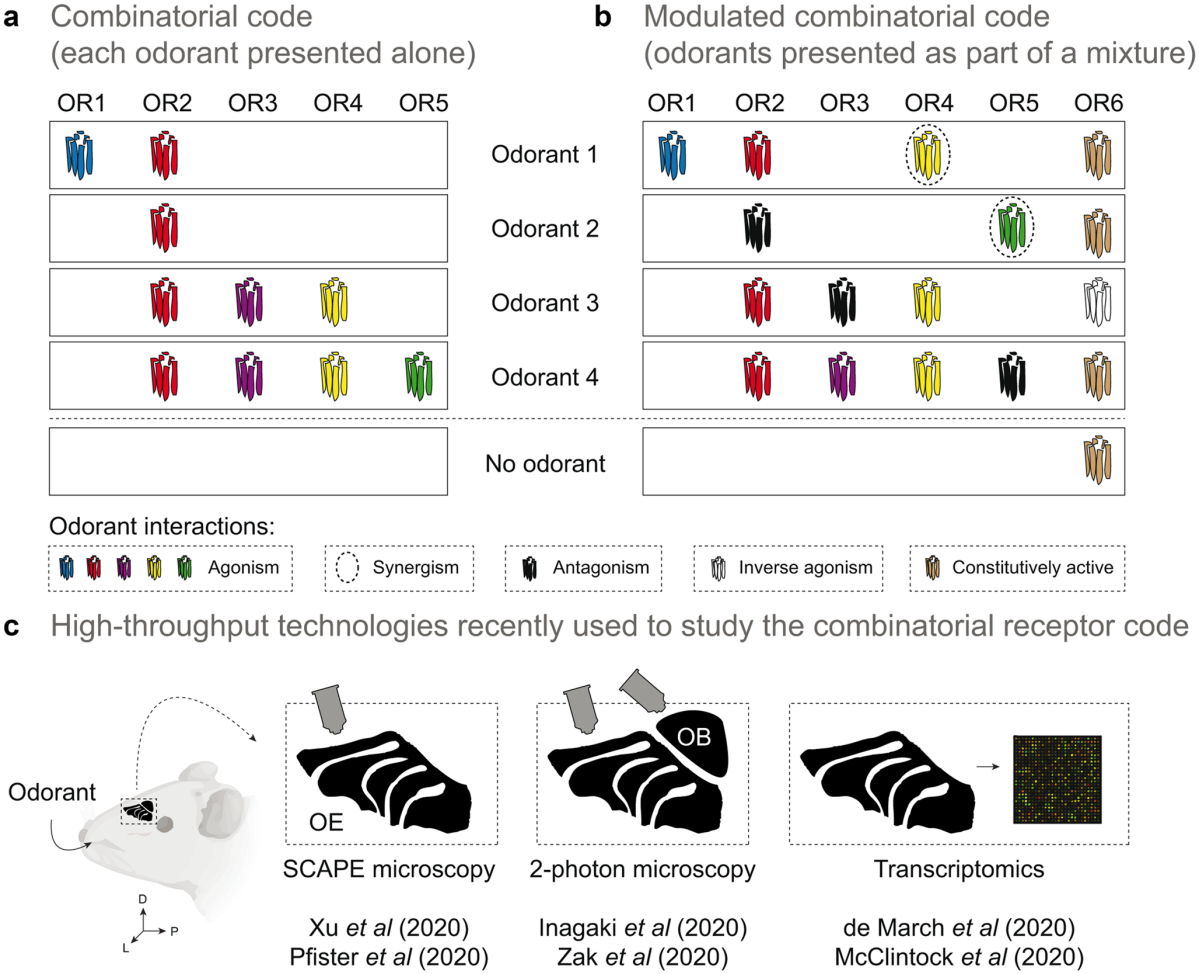
Modulation of the olfactory combinatorial code at the periphery. In the mammalian nose, odorants are detected by ORs in a combinatorial fashion. In other words, one odorant can activate multiple ORs, and each OR can detect more than one odorant. (a) When the combinatorial code was first established, each OR was tested against a given concentration of a single odorant, and only agonist interactions were analyzed. (b) Recent studies analyzed the responses of ORs to specific odorants presented as part of odor mixtures, and found that odorants in addition to their agonist role, can also serve as modulators (antagonists, inverse agonists, partial agonists and synergistic ligands) to OR activity. A *‘No odorant’* condition depicts the activation profile or ORs 1–6 in the absence of any odorant. (c) The recent studies mention above resorted to technologically advanced techniques to perform high-throughput analysis of OR/OSN activation in the OE, or the OSN axon terminals in the mouse OB, after exposure to odorants or odor mixes. Some of these studies resorted to 2-photon or Swept Confocally Aligned Planar Excitation (SCAPE) microscopy, while others used transcriptomic approaches (e.g., microarrays)

Initial experiments, recording from single OSNs in the OE, revealed that each OSN responds selectively to more than one odorant and that individual odorants activate unique sets of OSNs (Firestein, et al., 1993; Sato, et al., 1994; Sicard and Holley, 1984). In 1999, experiments combining calcium imaging and single-cell RT-PCR allowed, for the first time, the identification of the ORs expressed in OSNs – specifically OSNs activated by a group of aliphatic odorants which were tested at different concentrations (Malnic, et al., 1999). These results were revolutionary, as they provided concrete evidence that different odorants, or different concentrations of the same odorant, are recognized by a unique combination of multiple ORs. In other words, each concentration of a given odorant generates its own ‘*combinatorial receptor code’* in the OE (Fig. 3a).

Roughly a decade later, a study performed in the mouse OE the responses of 3000 dissociated mature OSNs to 125 different odorants, representing 375,000 possible OSN/OR-odorant pairings (Nara, et al., 2011). This large-scale study still constitutes the most comprehensive analysis of odorant response profiles in OSNs/ORs. It yielded three novel and significant findings about odor coding in the OE. First, OSNs/ORs repertoires exhibit an extraordinary diversity as well as bias, in odorant recognition. Second, most OSNs/ORs are narrowly tuned (i.e., detect one or a small number of structurally related odorants), although broadly tuned OSNs (i.e., responding to a large number of odorants) are also present in the OE. Third, the vast majority of odorants elicit a unique combinatorial activation code, containing both narrowly and broadly tuned OSNs/ORs.

Together, these results expanded our understanding of odorant detection and odor coding in the OE. In particular, these studies offered a potential explanation why different odorants exhibit distinct odors, including odorants that are structurally very similar yet diverge in sensory quality. Importantly, this combinatorial strategy deployed by OSNs, or ORs, has since been validated by many other studies and mammalian species (Araneda, et al., 2000, Araneda, et al., 2004, Duchamp-Viret, et al., 1999, Gonzalez-Kristeller, et al., 2015, Hamana, et al., 2003, Hu, et al., 2020, Jiang, et al., 2015, Kajiya, et al., 2001, McClintock, et al., 2014, Nara, et al., 2011, Oka, et al., 2004a, Oka, et al., 2004b, Saito, et al., 2009, Sato-Akuhara, et al., 2016, von der Weid, et al., 2015).

## Odor mixtures and odorant-OR interactions in the OE

Combinatorial coding helped to model the interaction of individual odorants with receptor repertoires. However, in natural environments, the olfactory system is exposed to complex mixtures of odorants, not to single odorants. Recent studies thus started to focus on mixture perception by investigating receptor responses to blends of multiple odorants (Fig. 3).

These experiments now shed a markedly new light on explanations of odor coding at the peripheral level. Specifically, results show that peripheral odor coding involves odorants acting on receptors as agonists, antagonists, inverse agonists, partial agonists, and even have a synergistic effect (Fig. 3b) (de March, et al., 2020; Inagaki, et al., 2020; McClintock, et al., 2020; Pfister, et al., 2020; Reddy, et al., 2018; Xu, et al., 2020; Zak, et al., 2020).

These advancements were fueled by technological progress (Fig. 3c). Notable here was the introduction of a new microscopy technique: Swept Confocally Aligned Planar Excitation (SCAPE). In a breakthrough study of the Firestein lab, SCAPE was used to test odor responses in OSNs of genetically engineered mice that expressed a calcium-sensitive fluorescent protein (GCaMP6f). Notably, this study measured odorant responses in intact epithelium tissue in real-time to analyze receptor responses in individual OSNs across a collection of ~ 10,000 cells (Xu, et al., 2020).

Observations of mixture coding yielded two notable effects: the suppression as well as the enhancement in individual cell responses. On the one hand, constituting suppression effects, responses to a mixture of three odorants (acetophenone, benzyl acetate, and citral) showed that some OSNs that responded predominantly to acetophenone when administered individually, had their responses to acetophenone suppressed or completely inhibited when exposed to the ternary odorant mixture. On the other hand, revealing enhancement effects, another subset of OSNs that initially yielded small responses to acetophenone and benzyl acetate alone, showed increased responses when a mix of the two odorants was added. Additionally, a group of OSNs that did not respond to citral showed a higher response to acetophenone when citral was included in the mix, which can be interpreted as a sign of synergistic effects. Synergistic effects were also found in another study, which analyzed responses to odorant mixtures via *in vivo* two-photon imaging of OSNs expressing GCaMP3. Here, the synergistic effects were seen preferentially for low concentrations of odorants in the mix. In comparison, antagonism was predominant for higher concentrations of odorants in the mixture (Inagaki, et al., 2020). These synergistic effects could indicate an allosteric mechanism, even though this type of modulation has rarely been reported for Class A GPCRs. Yet odorant binding to an allosteric site might explain, for example, why a receptor that did not respond to three individual odorants is active when exposed to a mixture of all three (Xu, et al., 2020).

A critical part of this revised model of odorant-receptor interaction is a markedly theoretical element. The SCAPE study presents a possible answer to the inherent neurocomputational challenge arising from combinatorial coding at the periphery: How does the brain discriminate different complex mixtures from widespread and overlapping receptor activity? Antagonistic modulation at the receptor level would facilitate sparse coding resulting in less ambiguous signal patterns. Aromatic blends, such as coffee or roses, are composed of hundreds of different components. The combinatorial code allows humans to detect various odorant features in such mixtures and respond to a complex and, in its constituents, unpredictable chemical environment. However, with combinatorial activation alone, receptor activation patterns quickly overlapped to form a broad and smudged signal, which would lose its distinctiveness. Modulation, antagonistic and allosteric, facilitates a unique receptor code for the discrimination of complex mixtures. Less, literally, can be more.

Another study used calcium imaging of dissociated mouse OSNs to analyze how indole sensitive cells respond to a mixture of indole and other odorants (Pfister, et al., 2020). The data demonstrated dose-dependent inhibition of the responses to indole by a variety of structurally diverse odorants. The ORs expressed by the indole responsive OSNs, as part of the same OR subfamily and additional OR paralogs, were identified by single-cell RNA-seq and characterized in a heterologous expression system. The activation profiles of these receptors to a large library of structurally diverse odorants (~ 800) showed that ~ 50% of the odorants in the library were able to antagonize at least one of these ORs, with some of them antagonizing one single OR and others antagonizing multiple ORs. Notably, the results indicate that antagonism by odorants may also occur in a combinatorial fashion. Overall, mathematical modeling of dose–response curves by antagonizing odorants remain consistent with competitive binding, while the authors do not exclude that some antagonist odorants could act non-competitively (allosterically) (Pfister, et al., 2020).

*In vivo* experiments in freely behaving mice indicated that responses of all indole sensitive ORs are inhibited when the agonist was mixed with α-ionone. In contrast, a different group of ORs proved responsive to the mixture (McClintock, et al., 2020). The same type of *in vivo* experiments, now followed by confirmation by *in vitro* expression of the ORs, demonstrated that the odorant whiskey lactone suppressed isoamyl acetate responses from a fraction of OSNs responsive to isoamyl acetate alone (de March, et al., 2020). Comparable antagonistic effects were found in binary mixtures containing undecanal and bourgenal. These experiments further registered inverse agonism, suppressing the receptors' basal activity upon exposure to bourgeonal, as well as partial agonism (de March, et al., 2020).

Antagonism proved the most common modulator of olfactory responses. However, the presence of inverse agonism (when an odorant binds to the receptor and decreases the receptor's basal activity) both *in vivo* and heterologous assays, remains notable (de March, et al., 2020; Inagaki, et al., 2020; Pfister, et al., 2020). An odorant can perform different causal roles, both as an agonist to one OR and an inverse agonist to another OR, as seen in heterologous essays (Inagaki, et al., 2020). These results are further consistent with previous observations (Ache, et al., 1988; Araneda, et al., 2004; Bell, et al., 1987; Chaput, et al., 2012; Duchamp-Viret, et al., 2003; Münch, et al., 2013; Oka, et al., 2004b; Peterlin, et al., 2008; Rospars, et al., 2008; Sanz, et al., 2005; Shirokova, et al., 2005; Spehr, et al., 2003).

Collectively these studies reveal that modulation of odorant responses can begin peripherally, at the OR level, before the stimulus reaches the olfactory bulb. Odorants act as agonists to activate ORs in a combinatorial fashion, but also serve as modulators (antagonists, inverse agonists, partial agonists and synergistic ligands) to OR activity (Fig. 3b).

Modulation of the combinatorial code points to two central explanatory tenets relevant to current research in olfaction. First, modulation explains how the olfactory system deals with the discrimination of overlapping receptor repertoires in high-dimensional odorant mixtures. Second, it links molecular data with psychophysical studies that have documented the psychological equivalent of suppression and enhancement effects in mixture perception (Cain, 1975; Kay, et al., 2005; Laing, et al., 1984).

## Other features of OR-ligand interactions

In addition to the efficacy of binding of the odorant to the OR and activation efficiency of the OR by the odorant, other mechanisms can further modulate OR/OSN responses, and consequently, odor perception. These perireceptor events include: odorant-odorant metabolic interaction (e.g., metabolic interaction among different aldehydes affect the olfactory metabolism of 2-methylbut-2-enal, a pheromone, and increase its availability in the OE), enzymatic conversion (e.g., reduction of hexanal to hexanol), dilution or removal of odor molecules by xenobiotic metabolizing enzymes in the nasal mucus (e.g., conversion of acetophenone to methyl salicylate by Cyp1a2a in the nasal mucus affects the response of Olfr874 to acetophenone) (Asakawa, et al., 2017; Hanser, et al., 2017; Ijichi, et al., 2019; Kida, et al., 2018; Nagashima and Touhara, 2010; Robert-Hazotte, et al., 2019; Thiebaud, et al., 2013), and were covered by multiple recent reviews (Block, 2018; Heydel, et al., 2013, 2019). Moreover, odorant-binding proteins, a class of lipocalin proteins secreted by nasal glands and capable of binding to odorant molecules, are present in vertebrates' mucus. However, their exact physiological role in olfaction still remains mostly unknown (Bignetti, et al., 1985; Briand, et al., 2002; Flower, 1996; Heydel, et al., 2013; Pevsner, et al., 1986; Tegoni, et al., 2000). Future work will help elucidate how these additional mechanisms further shape OR-ligand interactions and contribute to odor perception.

## Concluding remarks

How olfactory cues are translated into complex behavioral or physiological changes presents one of neuroscience's greatest mysteries. Odor coding at the periphery is central to solving this secret. Combinatorial coding and receptor modulation mechanisms reveal that odorant-binding at the periphery is not a passive interface that translates chemical data into electrical signals. These primary detection mechanisms actively structure the chemical data that reaches the brain. As Lettvin and colleagues had noted most famously for the visual system: the (frog's) retina performs computations in the first layer of input that renders the foundations for any subsequent signal computation model in the brain (Lettvin, et al., 1959). The same principle applies to olfaction. Therefore, it is imperative to model odor coding principles based on the odorant receptor's activity.

Odorant receptors are not ruled by the same mechanisms as the visual system. Odorants activate unique combinations of ORs in the OE. Some odorants can trigger a large number of ORs. Other odorants are less promiscuous and activate only a small number of ORs. The composition and number of ORs recruited can also vary with changes in odorant concentration. This initial information is thought to be subsequently processed and interpreted in the olfactory bulb and higher brain centers, leading to the different odor percepts. Mounting evidence now suggests that the first level of modulation of odorant responses already occurs in the OE. Interactions among various odorant components in a mixture can modulate and alter the combinatorial code to a particular odorant at the OR level, including concentration-dependent activity. These results bring a new level of complexity to olfactory coding, where a large number of receptors and odorants are involved and several possible combinations of odorants. The role of these modulatory interactions in odorant perception can be addressed in experiments that analyze how much exposure to odorant mixtures, compared to exposure to single odorants, can change olfactory-driven behaviors in mice.

Future experiments should consider these odorant interactions at the periphery and investigate remaining unanswered questions. Do some odorants covalently interact with receptors and modify their ligand binding sites? Or can odorants in a mixture interact one with another to produce new chemical structures? An additional factor to consider is the relative concentration of the odorant components within a scent. We know that while fragrances are composed of multiple odorants, some are predominant in concentration or intensity, or in volatility. According to the Laing limit (Laing and Francis, 1989; Laing, 1987), identifying single odorants in mixtures gets more difficult as the number of odorants increases. Recent research indicates that this limit may not link to a saturation of the receptor code but is caused by antagonistic effects.

These constitute relatively new and revolutionary insights. Experimental advances investigating concrete interactions of odorants and receptors were primarily driven by the recent advent of high-throughput technologies, such as discussed in this review (e.g., RNA-sequencing, SCAPE microscopy). The next years thus promise the generation of larger datasets in a markedly faster pace that will allows us to better model how receptor interactions shape odor coding. Combining these data with other large datasets from multidimensional behavioral phenotyping studies, chemoinformatics and deep learning, will facilitate the understanding of the functional impact of the combinatorial code and its modulation in odor-guided behavioral and physiological responses.

But the final frontier to cracking the olfactory code promises to be of a markedly theoretical nature, as recent studies on the odorant-receptor interactions foreshadow a paradigm shift. Thus far, the evidence gathered strongly suggests a first level of modulation of odorant responses through **linear and non-linear interactions** in the OE. Plus, recent studies with a medicinal chemistry approach have shown that OSNs do not categorize odorants as similar according to the principles of organic chemistry (Poivet, et al., 2016, 2018). In fact, they respond to features not even recognized by recent machine learning studies mapping odorants to perceptual categories, such as the recent study by Keller and colleagues (Keller, et al., 2017). As a result, any neural correlates of odor perception cannot be found by directly mapping chemical input structures onto neural activation patterns. Instead, the new task emerging is to determine the principles by which the olfactory system detects, encodes, permutates, and modulates its information – starting with the mechanisms of odor coding right at the periphery.
